# Insights into the genetic influences of the microbiota on the life span of a host

**DOI:** 10.3389/fmicb.2023.1138979

**Published:** 2023-08-03

**Authors:** Fang Zhang, Liying Wang, Jiayu Jin, Yulu Pang, Hao Shi, Ziyi Fang, Han Wang, Yujie Du, Yufan Hu, Yingchun Zhang, Xiaoyue Ding, Zuobin Zhu

**Affiliations:** ^1^Morphological Experiment Center, Xuzhou Medical University, Xuzhou, China; ^2^Xuzhou Engineering Research Center of Medical Genetics and Transformation, Key Laboratory of Genetic Foundation and Clinical Application, Department of Genetics, Xuzhou Medical University, Xuzhou, China; ^3^School of Anesthesiology, Xuzhou Medical University, Xuzhou, Jiangsu, China; ^4^School of Life Sciences, Xuzhou Medical University, Xuzhou, Jiangsu, China; ^5^School of Clinical Medicine, Xuzhou Medical University, Xuzhou, Jiangsu, China

**Keywords:** aging, *Escherichia coli*, *Drosophila melanogaster*, *Caenorhabditis elegans*, lifespan extension

## Abstract

*Escherichia coli* (*E. coli*) mutant strains have been reported to extend the life span of *Caenorhabditis elegans* (*C. elegans*). However, the specific mechanisms through which the genes and pathways affect aging are not yet clear. In this study, we fed *Drosophila melanogaster* (fruit fly) various *E. coli* single-gene knockout strains to screen mutant strains with an extended lifespan. The results showed that *D. melanogaster* fed with *E. coli purE* had the longest mean lifespan, which was verified by *C. elegans*. We conducted RNA-sequencing and analysis of *C. elegans* fed with *E. coli purE* (a single-gene knockout mutant) to further explore the underlying molecular mechanism. We used differential gene expression (DGE) analysis, enrichment analysis, and gene set enrichment analysis (GSEA) to screen vital genes and modules with significant changes in overall expression. Our results suggest that *E. coli* mutant strains may affect the host lifespan by regulating the protein synthesis rate (*cfz-2*) and ATP level (*catp-4*). To conclude, our study could provide new insights into the genetic influences of the microbiota on the life span of a host and a basis for developing anti-aging probiotics and drugs.

## 1. Introduction

With the rapid development of modern medical technology, society is increasingly aging. Improving healthy aging and searching for longevity substances have become current biomedical research priorities. In a report published in Cell in 2017, researchers screened 3,983 *Escherichia coli* (*E. coli*) mutants obtained from an *E. coli* single-gene knockout library using *Caenorhabditis elegans* (*C. elegans*) as a model. They investigated 29 mutants that significantly prolonged the lifespan of *C. elegans*. The results showed that colanic acid (CA) significantly prolonged the lifespan of *C. elegans* (Han et al., 2018). In 2019, based on the previously mentioned report, it was found that *E. coli* mutants can affect the growth and development of *C. elegans* through the balance between bacterial iron and reactive oxygen species (ROS) (Zhang et al., 2019). Hence, intestinal bacteria have provided a new direction in anti-aging research.

In humans, food digestion and absorption processes occur in the gut, where a considerable number of microorganisms exist. Being the largest and most complex micro-ecosystem in the human body, gut microbes and their metabolites regulate human health and play an essential role in bridging the diet and host gap. Recent studies have shown that fecal transplantation improves physiological functions and prolongs the life span in mice, suggesting that gut bacteria can influence the aging process (Barcena et al., 2019). However, gut bacteria are too complex and difficult to standardize. In this regard, it has been suggested that the genetic variation in gut microbes can influence the host’s viability and drive the host’s evolution. A study by Guo et al. published in the December 2019 issue of science suggested that to study the effects of gut flora metabolites on the host, it is essential to simplify the study population by starting with a single gut bacterium and subjecting it to multiple knockouts (Guo et al., 2019). *E. coli*, constituting about 0.1% of the human gut flora, is the most widely studied prokaryotic model organism and an important species in biotechnology and microbiology (Gao et al., 2014). Therefore, we screened for *E. coli* mutants with putative anti-aging effects on the host from essential gut bacteria (*E. coli*) using hosts and intestinal bacteria. We also explored how the bacterial mutant strains regulate aging, using the transcriptional changes in the intestinal bacteria that affect the host’s aging as an entry point.

*C. elegans* is a unique experimental system owing to its small size, rapid life cycle, transparency, and well-annotated genome. Most importantly, an estimated 60%–80% of human homologous genes exist in the nematode genome (Kaletta and Hengartner, 2006). The fruit fly, *Drosophila melanogaster*, is another vital model organism for studying aging (Piper and Partridge, 2018). Seventy-seven percent of human aging-related genes are also expressed in *Drosophila*, and their lifespan experiments date back to 1913. The following aging features make *Drosophila* a good model organism for studying aging: increased sleep fragment, impaired negative geotaxis, reduced autonomic flight and crawl function, reduced resting metabolic rate, and neurological and cardiac decline (Tamura et al., 2003; Gargano et al., 2005; Iliadi et al., 2012). Moreover, its simple culture and short lifespan facilitate high-throughput screening experiments.

Using high-throughput screening, we selected 25 *E. coli* mutant strains that have been shown to have anti-aging ability in *C. elegans* (Han et al., 2018), which were subjected to longevity and climbing experiments. Active and UV-inactivated bacteria were used in an attempt to detect any differences between the two. Combining the phenotypic and bioinformatic analyses, we further investigated the pathways related to lifespan extension, including the evolutionarily conserved insulin/insulin-like growth factor (IGF), the target of rapamycin (TOR), and germline signaling pathways (Guarente and Kenyon, 2000; Pletcher et al., 2002; Hahn and Denlinger, 2011; Partridge et al., 2011). We tried to lay the foundation for subsequent molecular mechanistic studies and provide a research basis for developing anti-aging probiotics and subsequent drugs.

## 2. Materials and methods

### 2.1. Experimental subjects and strains

*D. melanogaster* was selected from the Canton Special (CS) strain of wild-type *Drosophila* from the *Drosophila* Resource and Technology Platform, Center of Excellence in Molecular Cell Science, Chinese Academy of Sciences. *E. coli* single gene knockout strains were obtained from the single knockout library (*E. coli* Keio Knockout Collection). *C. elegans* strains were obtained from Caenorhabditis Genetics Center (CGC), University of Minnesota, United States. *C. elegans strains* were grown on a nematode growth medium (NGM) at 20°C. All flies were reared on standard cornmeal-yeast-agar medium at 25°C with a photoperiod of 12 h:12hLD (light:dark). *E. coli* was incubated in the Luria-Bertani Culture and used for the experiment when it reached OD_600_ = 1.

Ten-day post-adult male *Drosophila* were transferred to tubes containing an experimental growth medium grown (standard cornmeal-yeast-agar medium without yeast extract) at 25°C. Every 20 individuals were fed in one tube, in groups of four, with activated and inactivated *E. coli* of different mutant strains, focusing on mutant strains *purE*, *aroG*, and wild type *BW25113*. The experiment was repeated three times. During this process, each tube contains 200 μL experimental bacteria (OD_600_ = 1), which is replaced every 10 days. In addition, inactivated strains are obtained by UV irradiation of mutant strains for 30 min.

### 2.2. Lifespan assays

*C. elegans* and *Drosophila* lifespan assays are briefly described as follows. For the *C. elegans* lifespan assay, young adult worms were transferred to NGM containing *E. coli BW25113* and *purE*. Then, worm survival was measured daily by observing the pharyngeal movement and the touch-provoked movement using a platinum wire. Worms were considered dead if there was no pharyngeal and no touch-provoked movement. For the *Drosophila* lifespan assay, the second day of *Drosophila* tube entry was considered day one. The number of *Drosophila* deaths in each strain tube was recorded at 13:00 daily until they all died. Regarding the survival index, the mean lifespan is the mean of the number of days that all fruit flies in each tube survived; the maximum lifespan of each group is the lifespan of the fruit fly that last died; Survival was assessed every day based on the number of dead and alive flies; LT50 was calculated as the number of days it takes for 50% of the flies to die (Charalambous et al., 2022). The mean lifespan of *Drosophila* is calculated as follows. Let the number of days the fruit flies survived be “*d*,” and the number of flies that die on the same day be “*xd*.” Then, the mean lifespan of *Drosophila* per tube is


L=Σd∗xd/20

### 2.3. Behavioral assay

*Drosophila* climbing experiments for health span assays were performed as follows. The tubes of days 10 and 15 were placed vertically upside down at 18:00 daily. The number of fruit flies that reached the top of the tube in 10 s was examined after they had adapted, and the climbing process was recorded using a video camera for observation. The climbing index was calculated as follows: the number of fruit flies reaching the top of the tube within 10 s divided by the total number of alive fruit flies. Each tube was examined at least three times with at least 1 min apart. The average of the climbing index of each tube was recorded for each group.

### 2.4. *Caenorhabditis elegans* RNA-seq data analysis

In this study, three *C. elegans* fed with *E. coli purE* mutation samples and three *BW25113* control samples were sequenced on the DNBSEQ platform, averagely generating about 1.19 Gb per sample (BioProject: F21FTSECWLJ1283_NEMyjzwN). Raw reads with rRNA, low quality, joint contamination, and high content of unknown base nucleotides were filtered out. Then, clean reads were matched to the reference genome (NCBI: GCF_000002985.6_WBcel235) using HISAT and assembled using StringTie. Clean reads were compared to the reference sequence using the Bowtie 2 software. Differential gene expression between the *C. elegans* fed with *purE* and *BW25113* were analyzed by the Huada Online Analysis Software (Dr. Tom) using read count. The fold change >2 and *Q*-value <0.05 indicated significantly different gene expressions. The differentially expressed genes were mapped for gene function evaluation using the gene ontology (GO) database and the online analysis platform of Dr. Tom. They were also analyzed using the Kyoto Encyclopedia of Genes and Genomes (KEGG) enrichment analysis and Gene Set Enrichment Analysis (GSEA).

### 2.5. Statistical analysis

Statistical analyses were performed using student’s *t*-test (SPSS 19.0). Data were presented as mean ± standard deviation. Asterisks denote significant differences (*p < 0.05) as determined by student’s *t*-test. The anti-aging ability of the mutant strains was analyzed together with lifespan and health span (climbing index) assays. The survival curves of the *Drosophila* and *C. elegans* were analyzed with the Kaplan–Meyer method and the log-rank test.

## 3. Results

### 3.1. *Caenorhabditis elegans* and *drosophila* fed with *Escherichia coli purE* live longer than those fed with *Escherichia coli BW25113*

**Figure 1:**
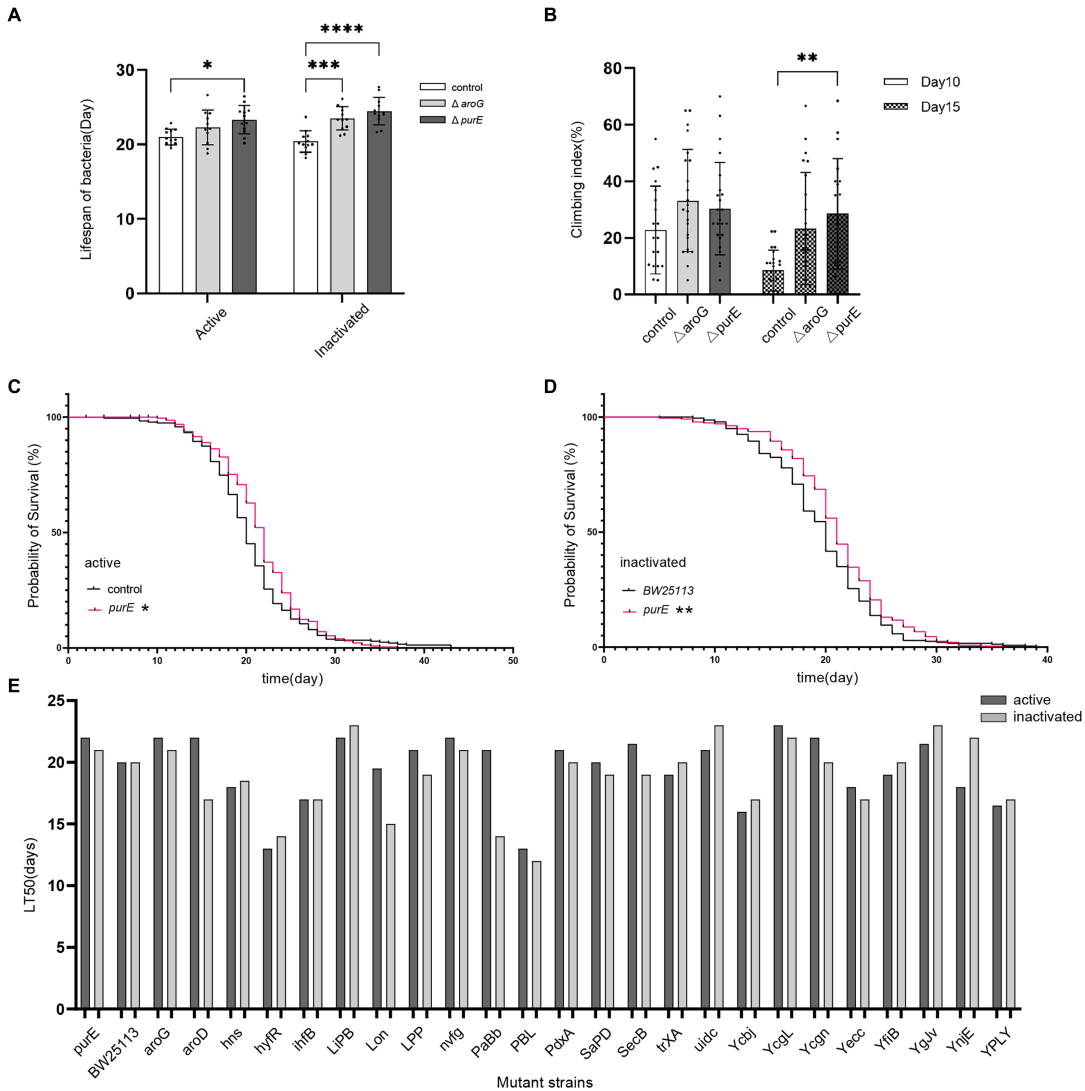
Comparison of the lifespan and climbing index of *Drosophila* fed with *E. coli* mutant strains *purE*, *aroG*, and *BW25113*. **(A)** Mean lifespan of *Drosophila* fed with *E. coli* mutant strains *purE*, *aroG*, and *BW25113*. **(B)** Days 10 and 15 of the climbing index of *Drosophila* fed *E. coli purE* compared with those fed with *BW25113*. * indicates significant differences, compared with *BW25113* (student’s *t*-test value of *p* <0.05). **, ***, and **** indicate student’s *t*-test value of *p* < 0.01, *p* < 0.001, and *p* < 0.0001, respectively. **(C)** Survival curves of *Drosophila* fed with active *E. coli purE* (red lines) and *BW25113* (black lines). **(D)** Survival curves of *Drosophila* fed with inactivated *E. coli purE* (red lines) and *BW25113* (black lines). * indicates significant differences, compared with *BW25113* (Log-rank test value of *p* < 0.05). **indicate Log-rank test value of *p* < 0.01.**(E)** The survival of *Drosophila* evaluated as the number of days it takes to reach 50% fly mortality (LT50) following feeding *E. coli* mutant strains.

**Figure 2:**
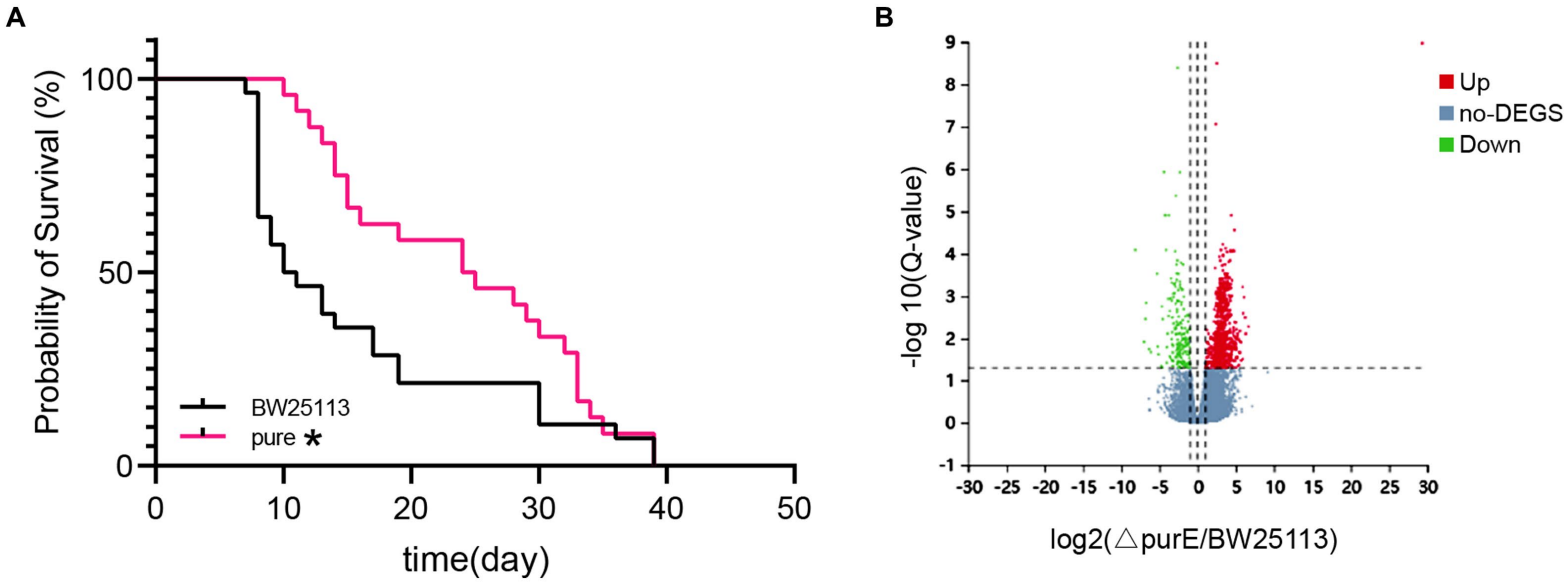
**(A)** Survival curve graph of *C. elegans* fed with *E. coli purE* and *BW25113*. **(B)** Volcano plot of the differential genes. * indicates significant differences, compared with *BW25113* (Log-rank test value of *p* < 0.05).

To test whether the different strains of *E. coli* extend the lifespan, we measured the mean lifespan of *D. melanogaster* fed with different *E. coli* mutant strains. The results showed that *Drosophila* fed with *E. coli purE* and *aroG* mutant strains had a significantly longer lifespan than those fed with *E. coli BW25113* (Supplementary Table S1, Table 1, and Figure 1A). *Drosophila* fed with *E. coli purE*, especially, had the longest mean lifespan, which was verified by *C. elegans* (Supplementary Table S1 and Figure 2A). The survival curves and half-lives of *Drosophila* fed with *E. coli purE* mutant strain showed a significant increase in host lifespan compared to the control group (*p* = 0.0212), as well as a higher median lifespan (LT50 = 22) (Supplementary Table S1 and Figures 1C–E). Meanwhile, the effects of live bacteria were more pronounced than that of inactivated bacteria, except for the value of *p*, but still significant. To better assess the health of flies with increased lifespan, day 10 and day 15 *Drosophila* climbing experiment data showed a significant increase in the climbing ability of *Drosophila* fed with *E. coli purE* mutant strain compared with those fed with *E. coli BW25113* (Figure 1B). The mean climbing index was higher than that of *Drosophila* fed with *E. coli BW*25113.

**Table 1:** Mean lifespan and rate of change in drosophila in three replicate experimental groups.

Strains	Active			Inactivated		
	No. of flies*	Mean + SD Lifespan (Day)**	Change (%)	No. of flies	Mean + SD Lifespan (Day)	Change (%)
BW25113	240	21.01 + 1.04	0	240	20.40 + 1.44	0
aroG	220	22.31 + 2.32	+6.21%	240	23.51 + 1.55	+15.24%
purE	240	23.32 + 1.91	+11.00%	240	24.47 + 1.85	+19.97%

*Includes three replicate groups, four parallel groups of 20 Drosophila per replicate. **The mean of each parallel group (20 animals) was counted by student’s *t*-test.

The longevity of *C. elegans* fed with *E. coli purE* and *BW25113* was also recorded, and survival curves were plotted (Figure 2A). There was a significant increase in longevity after feeding with *E. coli purE*.

### 3.2. Genetic analysis of *Caenorhabditis elegans* fed with *Escherichia coli purE* and *BW25113*

Gene expression analysis of *C. elegans* fed with lifespan-extending *E. coli* mutant strains was performed. We used differential gene expression analysis, KEGG pathway enrichment analysis, and GO enrichment analysis to analyze the sequencing data. GSEA analysis was performed to find genes associated with an extended lifespan.

The samples were analyzed in three independent biological replicates (six RNA-Seq libraries). Pearson’s correlation coefficient analysis showed high reproducibility between the three replicates, ranging from 0.91 to 0.99 (Table 2).

**Table 2:** Pearson correlation coefficient between two *C. elegans* replicate groups.

Sample	BW25113-1	BW25113-2	BW25113-3	purE-1	purE-2	purE-3
BW25113-1	1.00	0.99	0.95	0.99	0.91	0.96
BW25113-2	0.99	1.00	0.96	0.98	0.91	0.96
BW25113-3	0.95	0.96	1.00	0.97	0.91	0.98
purE-1	0.99	0.98	0.97	1.00	0.93	0.97
purE-2	0.91	0.91	0.91	0.93	1.00	0.96
purE-3	0.96	0.96	0.98	0.97	0.96	1.00

#### 3.2.1. DEGs and Go/KEGG enrichment analysis

The differentially expressed gene (DEG) analysis showed that there were 1,001 DEGs (*Q*-value <0.05) between *E. coli purE* and *BW25113* obtained by the mean read count. Among them, there were 810 upregulated genes and 191 downregulated genes of more than twofold in *E. coli purE*, as shown in the volcano plot (Figure 2B and Supplementary Table S2).

**Figure 3:**
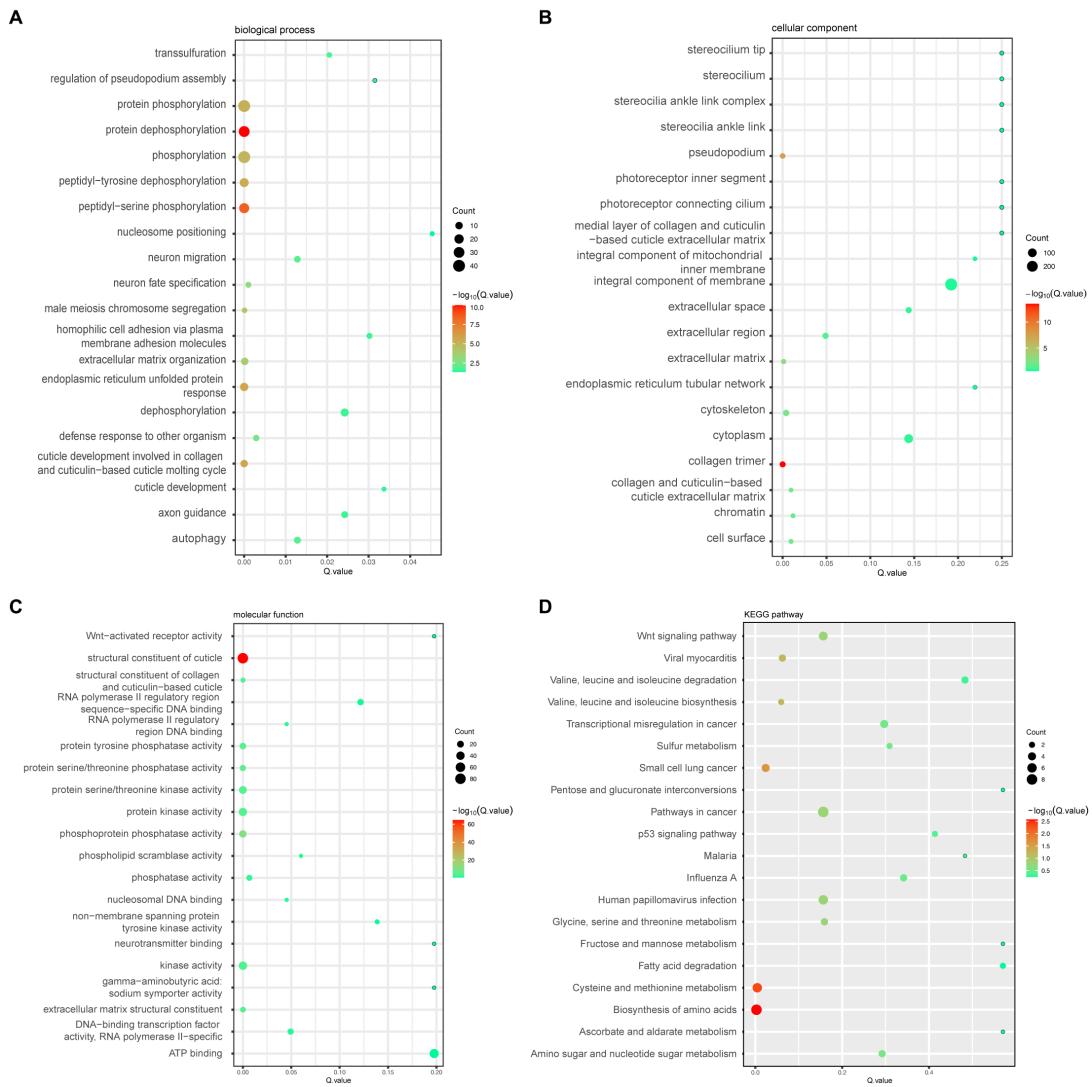
**(A)** Bubble plots of the top 20 biological processes enriched by the differential genes. **(B)** Bubble plots of the top 20 cellular components enriched by the differential genes. **(C)** Bubble plots of the top 20 molecular functions enriched by the differential genes. **(D)** Bubble plots of the top 20 KEGG pathways enriched by the differential genes.

To explore DEGs’ function and find whether they are associated with pathways known to extend the lifespan, we performed KEGG pathway and GO enrichment analyses. GO enrichment analysis showed that DEGs significantly enriched many biological processes, such as protein dephosphorylation, protein phosphorylation, and peptidyl-serine phosphorylation, and had significant effects on collagen trimer and the pseudopodium in the cellular component and the structural constituent of the cuticle in the molecular function (Figures 3A-C).

Meanwhile, the enrichment results of KEGG signaling pathways for the differential genes showed that the enriched genes were significantly enriched in the following pathways: the biosynthesis of amino acids, cysteine and methionine metabolism, small cell lung cancer pathway, and cancer pathways (Figure 3D). Additionally, we looked for signaling pathways in all the enriched pathways that are known to be associated with the longevity regulation pathway, where gene *cfz-2* was enriched in the mTOR signaling pathway (map04150) and gene *catp-4* was enriched in the insulin secretion pathway (map04911) (Table 3).

**Table 3:** KEGG pathway enrichment results in longevity regulation-related pathways and differential genes.

KEGG pathway term description	KEGG pathway term level1	KEGG pathway term level2	DEG	GeneBank description	Rich ratio *p*-value *q*-value		
Insulin secretion	Organismal systems	Endocrine system	catp-4	Sodium/potassium-transporting ATPase subunit alpha	0.033	0.398	0.611
mTOR signaling pathway	Environmental information processing	Signal transduction	cfz-2	Frizzled-2	0.013	0.735	0.771

#### 3.2.2. GSEA resulted in pathways and modules whose overall expression levels were upregulated or downregulated

To avoid missing genes that are not significantly differentially expressed but are biologically important, the experiment was performed simultaneously with GSEA of all genes. GSEA resulted in 13 upregulated pathways, 38 downregulated pathways (*Q*-value <0.05), and 3 upregulated modules (value of *p* <0.05; *Q*-value <0.25) for the overall expression level. The set of genes contained in the pathway was highly expressed in the *purE* or *BW25113* group, and the details of the top 10 are shown in Table 4.

**Table 4:** Details of pathways and modules with significant changes in overall expression level by GSEA.

Term description	Category	up/down	KEGG pathway term level1	Size	ES	*p*-value	*q*-value
Oxidative phosphorylation	Pathway	up	Metabolism	95	0.520	0.00E+00	0.00E+00
Glycine, serine and threonine metabolism	Pathway	up	Metabolism	27	0.621	0.00E+00	1.85E−03
Parkinson disease	Pathway	up	Human Diseases	95	0.480	0.00E+00	2.42E−03
Arginine and proline metabolism	Pathway	up	Metabolism	26	0.600	0.00E+00	5.74E−03
Biosynthesis of amino acids	Pathway	up	Metabolism	72	0.449	0.00E+00	1.94E−02
Alzheimer disease	Pathway	up	Human Diseases	110	0.407	0.00E+00	2.08E−02
Cardiac muscle contraction	Pathway	up	Organismal Systems	30	0.523	1.69E−03	2.15E−02
Butanoate metabolism	Pathway	up	Metabolism	19	0.580	3.38E−03	2.18E−02
Glutathione metabolism	Pathway	up	Metabolism	50	0.473	1.59E−03	2.32E−02
Cysteine and methionine metabolism	Pathway	up	Metabolism	42	0.502	1.62E−03	2.42E−02
Spliceosome	Pathway	down	Genetic Information Processing	112	−0.499	0.00E+00	0.00E+00
Endocytosis	Pathway	down	Cellular Processes	103	−0.488	0.00E+00	0.00E+00
mRNA surveillance pathway	Pathway	down	Genetic Information Processing	67	−0.538	0.00E+00	0.00E+00
Cell cycle	Pathway	down	Cellular Processes	82	−0.502	0.00E+00	0.00E+00
Hippo signaling pathway	Pathway	down	Environmental Information Processing	57	−0.515	0.00E+00	2.94E−04
Breast cancer	Pathway	down	Human Diseases	38	−0.565	0.00E+00	4.79E−04
Autophagy – other	Pathway	down	Cellular Processes	24	−0.653	0.00E+00	5.39E−04
Transcriptional misregulation in cancer	Pathway	down	Human Diseases	49	−0.537	0.00E+00	6.16E−04
Fanconi anemia pathway	Pathway	down	Genetic Information Processing	25	−0.619	0.00E+00	8.62E−04
RNA transport	Pathway	down	Genetic Information Processing	116	−0.430	0.00E+00	9.06E−04
Lysine degradation	Module	up	/	15	0.612	1.74E−03	6.09E−02
F-type ATPase, eukaryotes	Module	up	/	15	0.572	1.85E−02	7.50E−02
V-type ATPase, eukaryotes	Module	up	/	20	0.495	2.40E−02	1.09E−01

**Figure 4:**
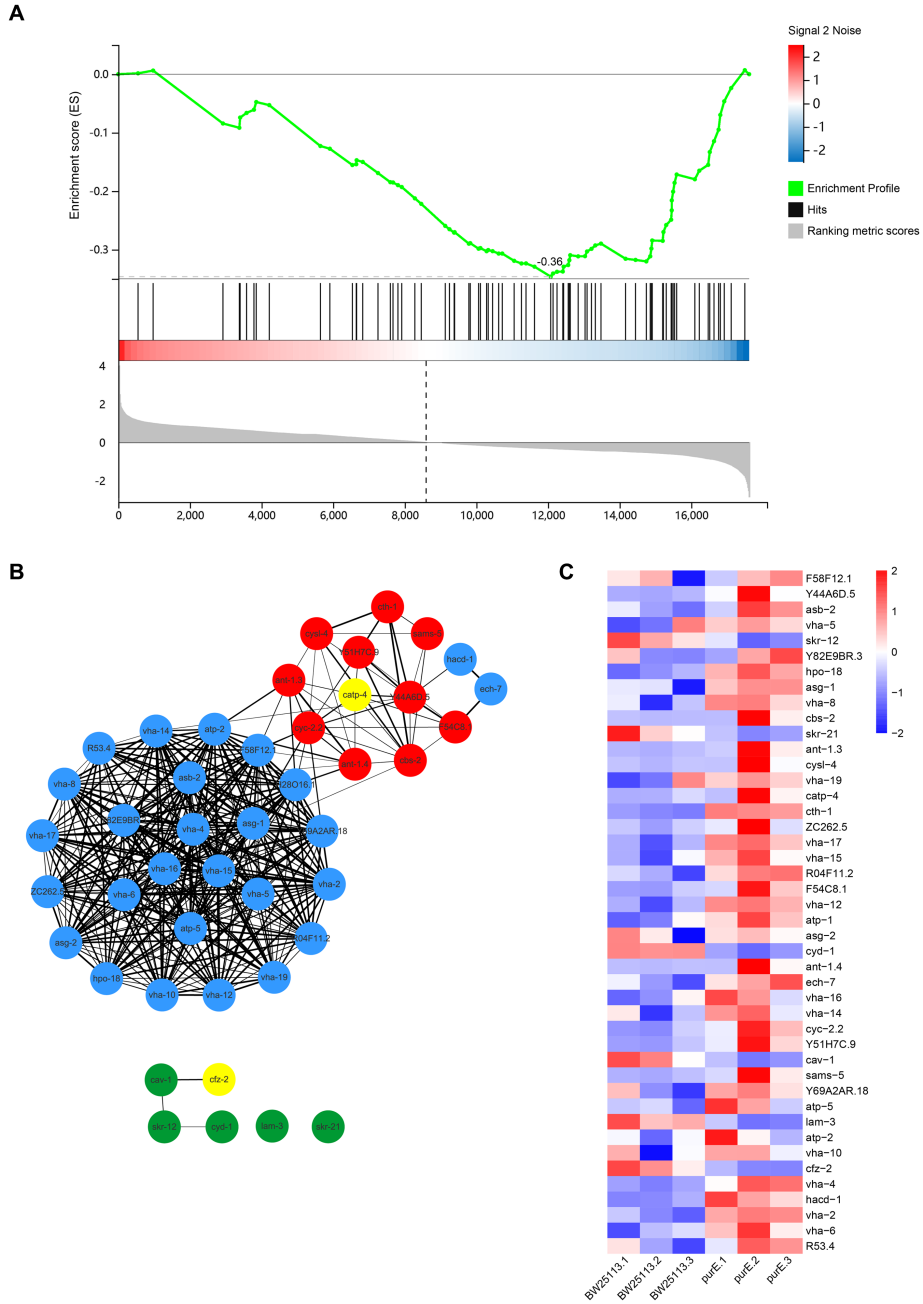
**(A)** GSEA plot of the mTOR signaling pathway. All genes are arranged according to decreasing differential expression ploidy. Genes are heavily enriched downstream. Enrichment score (ES) = −0.36; size = 78; subsets gene number = 39; normalized enrichment score (NES) = −1.66; nominal value of *p* = 5.88e−3; FDR *Q*-value = 0.05. **(B)** Network interactions of meaningful genes in GSEA. The red circles in the graph are upregulated by a factor of 2 or more DEGs. The green circles are DEGs downregulated by a factor of 2. The yellow circles indicate genes associated with known lifespan-related pathways. The thickness of the line represents the score between genes. The minimum required interaction score is a medium confidence level (0.400). **(C)** Heat map of meaningful genes in GSEA.

Note that the mTOR signaling pathway is also enriched here (Figure 4A), and the results show that the overall expression of the pathway is downregulated.

In the GSEA, the core genes that made significant contributions to the enrichment score were selected. Sixteen differentially expressed genes, including *cfz-2* and *catp-4*, were obtained in 3.2.1, and there are 28 core genes in 3 modules. We constructed the reciprocal network of these genes using STRING and Cytoscape 3.9.0 (Figure 4B) and mapped the expression heat map (Figure 4C). The results showed a stronger association between the upregulated genes and the other genes than the downregulated genes. By looking at the heat map, one can see some differential genes between the two groups.

## 4. Discussion

It has been shown that *purE* knockout *E. coli* can extend the lifespan of *C. elegans* by 21% compared with *BW25113* wild type (Han et al., 2018). We studied the effects of 25 *E. coli* mutant strains, including *purE*, on lifespan and locomotion in *Drosophila* based on this provided list, and determined the anti-aging properties of *PurE E. coli* mutant on its host. The gene *purE* is N (5)-carboxyaminoimidazole ribonucleotide mutase, belonging to *E. coli str. K-12 substr*. *PurE* was previously thought to be the catalytic subunit of phosphoribosylaminoimidazole carboxylase, with ATPase subunit PurK (Tiedeman et al., 1989).

Studies have shown that diet is an influential factor in the lifespan of *Drosophila*. For example, dietary restrictions can reduce mortality in *Drosophila* at specific ages (Mair et al., 2003; Rizza et al., 2014), and changing the proportion of nutrients consumed affects their lifespan (Mair et al., 2005; Lee et al., 2008; Skorupa et al., 2008; Grandison et al., 2009; Solon-Biet et al., 2020). Moreover, this experiment further confirmed that feeding *Drosophila* with *E. coli purE* extended their lifespan. The mean lifespan could be extended by more than 10%, and the climbing ability was correspondingly improved, showing a better anti-aging ability. Notably, compared to live bacteria, UV-inactivated bacteria induce increase survival rate and average lifespan of the host. We reasoned that it may be because the mutant bacteria under UV stimulation activated the stress mechanism and produced some active substances that are beneficial for longevity, just like the discovery of active yeast derivatives (Levin, 1998). Then, to further explore the mechanism of *PurE*-dependent lifespan extension in flies and nematodes, we performed sequencing analysis of nematodes fed with strain *purE* and wild type *BW25113*, respectively.

After performing bioinformatics analysis, 1,001 DEGs with more than twofold significant changes were obtained (FDR *Q* < 0.05), including 810 upregulated DEGs and 191 downregulated DEGs. After performing an enrichment analysis of DEGs, the following nutritional signaling pathways that have been shown to extend lifespan were identified: insulin/*IGF-1* signaling, which maintains glucose homeostasis; mTOR signaling, which senses and transmits amino acid signals; GCN2/ATF4. The following genes and pathways were found to be valuable for the follow-up studies, which may help us identify the anti-aging active substances produced by *E. coli* and the molecular mechanisms behind their action. Among them, two differential genes are of interest, although the scores were not significant when KEGG was enriched, that may be because the number of differential genes was large so the significance was reduced, but these two genes have strong research significance due to their close relationship with longevity-related pathways, so they are taken out for discussion. In addition, we also used GSEA to screen for additional missing genes and modules. Overall, these bioinformatic analyses focused two longevity related genes for us: *cfz-2* and *catp-4*, which may be potential genes to extend lifespan.

DEG *cfz-2* was found to be enriched in the pathway mTOR signaling pathway and its associated longevity regulatory pathway. *Cfz-2* expression was downregulated by 2.08 fold in the *PurE*-treated worms, while the *cfz-2* gene is conserved in humans. The *C. elegans* Frizzled *cfz-2* has been reported to be involved in cell migration, neuronal migration, and positive regulation of motor neuron migration and is required for cell migration and interacts with multiple Wnt signaling pathways (Zinovyeva and Forrester, 2005). *cfz-2* may non-autonomously direct cell migrations, whereas the Wnt gene *CWN-2* may act through *cfz-2* for specific cell migrations (Bhanot et al., 1996; Lin et al., 1997; Hsieh et al., 1999; Dann et al., 2001). In addition, we found that *cfz-2* is enriched in the mTOR signaling pathway through KEGG analysis, which may be because the Wnt signaling pathway is one of the upstream signaling pathways of mTOR. Thus, a substantial reduction in *cfz-2* expression may further affect the host’s lifespan by affecting Wnt and mTOR signaling pathway-related cell migrations.

Another DEG is *catp-4*, which is enriched in the insulin secretion pathway. *Catp-4* expression is upregulated by 2.78 fold in the *PurE*-treated worms. Catp-4 was predicted to enable P-type sodium: potassium-exchanging transporter activity and be an integral component of the membrane. It is in direct lineage with human *ATP12A* and *ATP4A*. The Alliance of Genome Resources mentions that *catp-4* regulates potassium-sodium ion homeostasis. This may provide us with an idea for subsequent studies focused on the fact that *catp-4* may further regulate ATP levels *in vivo* by regulating ion homeostasis and ion transport in the cell membrane, thereby affecting the host’s lifespan.

Furthermore, to avoid missing the regulation of non-differential genes in the overall expression level of the pathway, GSEA further obtained critical pathways and modules for the overall expression level changes including the following: oxidative phosphorylation; glycine, serine, and threonine metabolism; spliceosome; endocytosis; autophagy; upregulation of the overall expression of lysine degradation module and F-type/V-type ATPase module. The changes in these pathways and modules may significantly contribute to lifespan regulation. For example, the V-type ATPase module contains all VHA genes. The putative V-type proton ATPase is involved in the positive regulation of programmed cell death and developmental apoptosis. The vacuolar (H+) ATPase is involved in the positive regulation of oocyte development and protein-targeted membranes. These are important candidate genes for further studies on anti-aging mechanisms.

## Data availability statement

The datasets presented in this study can be found in online repositories. The names of the repository/repositories and accession number(s) can be found at: https://www.ncbi.nlm.nih.gov/geo/, GSE214285.

## Ethics statement

Ethical review and approval was not required for the study on animals in accordance with the local legislation and institutional requirements.

## Author contributions

FZ, LW, and JJ have contributed equally to this work and responsible for the conceptualization, methodology, visualization, writing, reviewing, editing, and funding acquisition. HS, ZF, YP, and HW were responsible for the methodology, software, validation, and investigation. YD, YH, and YZ were responsible for the formal analysis. XD and ZZ were responsible for the ideas, writing, reviewing, editing, and project administration. All authors contributed to the article and approved the submitted version.

## Funding

This work was supported by the National Natural Science Foundation of China (81701390), the Natural Science Foundation of Jiangsu Province (BK20170250), the Xuzhou Science and Technology Innovation Project (KC19057), the Jiangsu Postgraduate Innovation Program (730221059), and the Jiangsu University Students Innovation and Entrepreneurship Training Program (202010313026Z).

## Conflict of interest

The authors declare that the research was conducted in the absence of any commercial or financial relationships that could be construed as a potential conflict of interest.

## Publisher’s note

All claims expressed in this article are solely those of the authors and do not necessarily represent those of their affiliated organizations, or those of the publisher, the editors and the reviewers. Any product that may be evaluated in this article, or claim that may be made by its manufacturer, is not guaranteed or endorsed by the publisher.
