# Supplementary material for: Insights into the genetic influences of the microbiota on the life span of a host
Source: Front Microbiol. 2023 Aug 3;14:1138979. doi: 10.3389/fmicb.2023.1138979 (PMC10434519; doi:10.3389/fmicb.2023.1138979)
Supplement: Supplementary file 1 [file Table_1.docx]

| **Mutant strains** | **Average lifespan** | | **LT50(days)** | | **Logrank-p-value** | |
| --- | --- | --- | --- | --- | --- | --- |
|  | **Living** | **Inactivated** | **Living** | **Inactivated** | **Living** | **Inactivated** |
| purE | 23.32 | 24.47 | 22 | 21 | 0.0212 | 0.0059 |
| BW25113 | 21.01 | 20.40 | 20 | 20 | / | / |
| aroG | 22.31 | 23.51 | 22 | 21 | 0.0005 | <0.0001 |
| aroD | 16.80 | 17.30 | 22 | 17 | 0.0159 | <0.0001 |
| hns | 18.10 | 17.90 | 18 | 18.5 | <0.0001 | <0.0001 |
| hyfR | 13.60 | 14.90 | 13 | 14 | <0.0001 | <0.0001 |
| ihfB | 17.40 | 17.80 | 17 | 17 | <0.0001 | <0.0001 |
| LiPB | 21.95 | 23.93 | 22 | 23 | 0.0119 | <0.0001 |
| Lon | 16.91 | 18.25 | 19.5 | 15 | 0.2160 | <0.0001 |
| LPP | 21.40 | 19.80 | 21 | 19 | 0.5231 | 0.3981 |
| nvfg | 21.30 | 20.20 | 22 | 21 | 0.0835 | 0.1561 |
| PaBb | 16.42 | 17.79 | 21 | 14 | 0.0469 | <0.0001 |
| PBL | 16.10 | 13.90 | 13 | 12 | <0.0001 | <0.0001 |
| PdxA | 21.40 | 20.00 | 21 | 20 | 0.3339 | 0.7663 |
| SaPD | 19.80 | 19.10 | 20 | 19 | 0.2247 | 0.0383 |
| SecB | 22.00 | 19.50 | 21.5 | 19 | 0.0610 | 0.1816 |
| trXA | 21.19 | 23.46 | 19 | 20 | 0.4589 | 0.2818 |
| uidc | 22.46 | 23.24 | 21 | 23 | 0.0097 | <0.0001 |
| Ycbj | 17.30 | 17.40 | 16 | 17 | <0.0001 | <0.0001 |
| YcgL | 22.26 | 22.58 | 23 | 22 | 0.0023 | <0.0001 |
| Ycgn | 22.70 | 19.90 | 22 | 20 | 0.0005 | 0.8729 |
| Yecc | 17.60 | 16.90 | 18 | 17 | <0.0001 | <0.0001 |
| YfiB | 19.40 | 19.80 | 19 | 20 | 0.0028 | 0.5108 |
| YgJv | 20.90 | 23.80 | 21.5 | 23 | 0.3378 | <0.0001 |
| YnjE | 18.35 | 17.90 | 18 | 22 | 0.6263 | 0.0008 |
| YPLY | 17.50 | 16.50 | 16.5 | 17 | <0.0001 | <0.0001 |

**Table S1** Lifespan of *Drosophila melanogaster* fed with 25 *E. coli* mutant and wild-type strains

The table shows the lifespan of *D. melanogaster* after feeding 25 knockout *E. coli* and wild-type strains at 29°C. These 25 E. coli mutants were selected from Han et al (PMID: 28622510). The results show that the *purE* mutant strains better enhance the lifespan of *D. melanogaster* compared to the wild-type, both in the living and inactivated state of *E. coli*. In this case, the *purE*, *aroG* and *BW25113* strains all completed three replicate experiments, with four parallel groups of 20 flies each and the mean calculated. The other 23 groups of mutant strains each completed four experiments with 20 individuals in each group.
